# Nomogram for prediction of gestational diabetes mellitus in urban, Chinese, pregnant women

**DOI:** 10.1186/s12884-019-2703-y

**Published:** 2020-01-20

**Authors:** Fei Guo, Shuai Yang, Yong Zhang, Xi Yang, Chen Zhang, Jianxia Fan

**Affiliations:** 1 The International Peace Maternity and Child Health Hospital, School of Medicine, Shanghai Jiao Tong University, Shanghai, China; 2 Shanghai Key Laboratory of Embryo Original Diseases, Shanghai, China; 3 Shanghai Municipal Key Clinical Specialty, Shanghai, China; 4 Obstetrics Department, The International Peace Maternity & Child Health Hospital The China Welfare Institute, 910 Hengshan Rd, Shanghai, China

**Keywords:** Body mass index, Prediction model, Pregnancy, Gestational diabetes mellitus, Nomogram

## Abstract

**Background:**

This study sought to develop and validate a nomogram for prediction of gestational diabetes mellitus (GDM) in an urban, Chinese, antenatal population.

**Methods:**

Age, pre-pregnancy body mass index (BMI), fasting plasma glucose (FPG) in the first trimester and diabetes in first degree relatives were incorporated as validated risk factors. A prediction model (nomogram) for GDM was developed using multiple logistic regression analysis, from a retrospective study conducted on 3956 women who underwent their first antenatal visit during 2015 in Shanghai. Performance of the nomogram was assessed through discrimination and calibration. We refined the predicting model with t-distributed stochastic neighbor embedding (t-SNE) to distinguish GDM from non-GDM. The results were validated using bootstrap resampling and a prospective cohort of 6572 women during 2016 at the same institution.

**Results:**

Advanced age, pre-pregnancy BMI, high first-trimester, fasting, plasma glucose, and, a family history of diabetes were positively correlated with the development of GDM. This model had an area under the receiver operating characteristic (ROC) curve of 0.69 [95% CI:0.67–0.72, *p* < 0.0001]. The calibration curve for probability of GDM showed good consistency between nomogram prediction and actual observation. In the validation cohort, the ROC curve was 0.70 [95% CI: 0.68–0.72, *p* < 0.0001] and the calibration plot was well calibrated. In exploratory and validation cohorts, the distinct regions of GDM and non-GDM were distinctly separated in the t-SNE, generating transitional boundaries in the image by color difference. Decision curve analysis showed that the model had a positive net benefit at threshold between 0.05 and 0.78.

**Conclusions:**

This study demonstrates the ability of our model to predict the development of GDM in women, during early stage of pregnancy.

## Background

Gestational diabetes mellitus (GDM) is a glucose intolerance condition that is first detected during pregnancy. Rapid societal transition from traditional foods and lifestyle to an obesogenic environment has exposed the Chinese people to high prevalence of GDM (8.1 to 19.7%) [1–3]. GDM caused by varying degrees of insulin resistance to placenta-derived hormones, and, which in turn, increases the maternal adipose tissue [4]. Diagnosis of GDM is usually conducted with an abnormal oral glucose tolerance test (OGTT) at 24-28th week of gestation. Recently, there has been a controversy that skipping glucose on the first prenatal visit, could lead to correct GDM diagnosis [5]. GDM has been reported to increase the risk of adverse fetal outcomes such as fetal macrosomia, and subsequent maternal consequences like metabolic syndrome and cardiovascular morbidity. However, early identification of identifying women at risk of GDM could aid in averting such risks, through early interventions [6, 7]. Various groups have attempted to develop models that can predict an abnormal OGTT occurrence at 24–28 weeks, based on risk factors identified in the first trimester. Methods such as scroring systems, glucose biochemical assays of, and glycosylated haemoglobin (HbA1c) levels have used in different populations with varying degrees of success [8–11]. Established risk factors of GDM include advanced maternal age, excessive weight gain during pregnancy, overweight or obesity, diabetes in first degree relatives, and giving birth to an infant with a macrosomia, etc [12] However, clinical trials have shown that GDM can be relatively prevented through an intensive lifestyle modification implemented before 20 weeks of gestation [13, 14].

Different populations have different risk profiles for GDM. In Western countries, GDM mainly occurs in obese women (body mass index [BMI] > 30 kg/m^2^) or women with increasing gestational weight gain [15–17]. In Asian countries however, it cuts across the women populations, making it difficult to pinpoint a possible cause. In the present study, our aim was to create a simple and implementable strategy for identifying GDM in pregnant Chinese women living in urban area.

## Methods

### Study design

This study conducted retrospectively from January 2015 to December 2015, in Shanghai. Dataset was obtained from the International Peace Maternity and Child Care Health Hospital (IPMCH) electronic medical record system. Eligible subjects who underwent first-trimester screening at the IPMCH were recruited in the study. The prospective cohort study was conducted in 2016. Demographic information of the subjects which included; last menstrual period, maternal age, gestational age, pre-pregnancy weight, current height, personal history, educational levels, parity, diabetes in first degree relatives, were obtained through a face-to-face interview questionnaire, at the first prenatal visit (9–13 weeks’ gestation). Pre-pregnancy BMI was calculated by dividing the pre-pregnancy weight (kg) by the height squared (m^2^) and classified based on the Chinese criteria (underweight, < 18.5 kg/m^2^; normal weight, < 24.0 kg/m^2^; overweight, 24.0 kg/m^2^ ≤ BMI < 28.0 kg/m^2^; obese, BMI ≥ 28.0 kg/m^2^) [18]. Venous blood samples were obtained after overnight fasting for analysis of FPG and HbA1c, at the first visit to antenatal clinic.

GDM was diagnosed at 24–28 weeks of gestation according to the American Diabetes Association (ADA) criteria using abnormal plasma glucose values during the 2-h, 75-g OGTT. Abnormal values were defined according to ADA thresholds: a fasting level of 5.1 mmol/l or greater, a 1-h value of 10.0 mmol/l or greater, and, a 2-h value of 8.5 mmol/l or greater.

### Exploratory cohort

The hospital-based retrospective cohort study included 4774 women, who underwent their initial prenatal visit (9–13 gestational weeks) at the IPMCH in 2015. Women with pre-existing diabetes (FPG ≥ 7 mmol/L or HbA1c ≥ 6.5% during the first antenatal care or self-reported previous diabetes) (*n* = 54), multifetal pregnancies (*n* = 215) and missing data (*n* = 214 for no FPG tested, *n* = 178 for no OGTT performed, *n* = 157 for no records on family history) were excluded from the study. The remaining 3956 eligible women were used in further analysis.

### External validation cohort

Eligible participants, who attended prenatal care in the first trimester with single pregnancy, were selected and followed-up until GDM diagnosis in 2016, at the IPMCH. Women with pre-existing diabetes were excluded (*n* = 292). Therefore, the 6572 OGTT-tested women formed the dependent validation cohort of this study.

### Development of an individualized prediction model

Risk factors for GDM include advanced age, high pre-pregnancy BMI, diabetes in first degree relatives and high FPG in the first trimester. In the exploratory cohort, multiple logistic regression analysis was used to estimate the coefficients of each risk factor and mutually-adjusted odds ratio (OR) assigned for GDM. The continuous predictor variables such as age, BMI and FPG were found to be linear with log odds of the outcomes. To provide clinicians with a quantitative tool to visually predict individual probability of GDM, we developed a nomogram based on multivariable logistic analysis in the exploratory cohort. To further quantify the accuracy of the prediction models in discriminating subjects with GDM from subjects without, a receiver operating characteristic (ROC) curve was plotted, and the area under the curve (AUC) was calculated. Calibration was evaluated with the calibration curve. Ideally, the closer the dots are to the 45 degree line the better the model. Internal validation was initially analyzed by bootstrapping with 1000 random samples drawn with replacement. The ROC curve and calibration plot for recalibrated train model were extended to internal and external validation datasets. Decision curve analysis was used to determine the clinical usefulness of the nomogram by quantifying the net benefits at different threshold probabilities in the validation datasets. Spatially mapped t-distributed stochastic neighbor embedding (t-SNE) was used to distinguish non-GDM from GDM, i.e. the ability to screen for a true negative group.

### Statistical analysis

Analyses were performed using SPSS version 23.0 (SPSS, Inc., Chicago, IL) and R statistical software version 3.6.1 (packages rms, rmda, and Rtsne). Continuous variables were presented as the means with standard deviations, while categorical data were expressed as counts and percentages. Levene’s test was used to determine the homogeneity of the variances, and, Kolmogorov-Smirnov was used to assess the normal distribution. Summary statistics between both groups were compared using either unpaired Student’s t-test or Mann-Whitney tests for continuous data, and chi-squared tests for categorical data. Crude and mutually adjusted OR with 95% confidence interval (CI) for associations between baseline risk factors and GDM were estimated using the logistical regression model. Nomogram and calibration curve were performed with the “rms” package. The package “rmda” provided tools for evaluating the value of using a risk prediction instrument in deciding treatment or intervention. The “Rtsne” package was used to assess the screening ability based on construction of a low dimensional embedding of high-dimensional data, distances or similarities. A *p*-value of < 0.05 was considered to indicate statistical significance.

## Results

### Baseline characteristics for two cohorts

In the retrospective cohort of 3956 women, 662 developed GDM (16.7% incidence). The median ± SD pregnancy week for the first prenatal visit was 10.2 ± 3.5. Their mean age was 30.61 years, mean pre-pregnancy BMI (21.45 kg/m^2^), median FPG value (4.45 mmol/L), and presence of diabetes in first degree relatives (27.4%). In the external validation cohort of 6572 women, 739 developed GDM (11.2% incidence). The median ± SD pregnancy week for the first prenatal visit was 10.4 ± 3.3. Their mean age was 30.84 years, mean pre-pregnancy BMI was (21.20 kg/m^2^), median FPG (4.40 mmol/L), and presence of diabetes in first degree relatives (16.5%). Comparison of baseline characteristics between GDM and non-GDM in the two cohorts is shown in Table 1. In both cohorts, women who developed GDM had a relatively higher prevalence of family history of diabetes, advanced age, increased pre-pregnancy BMI, FPG, and multipara. However, education levels of the two groups was similar (*p* > 0.05), as was fetal sex. Significant differences were observed in the clinical characteristics between the exploratory dataset and the validation dataset (*p* = 0.04 for age, 0.24 for BMI, 0.001 for family history and 0.03 for FPG). However, the key difference between the two cohorts was in the GDM incidence (16.7% v 11.2%, *p* < 0.001). Even though many factors could contribute to this difference in incidence, we attributed the scenario to an antenatal education program that teaching pregnant women to manage their diets and weight during pregnancy.

**Table 1:** General characteristics of women with normal glucose tolerance and those who developed GDM

	Retrospective cohort (*n* = 3956)			Prospective cohort (*n* = 6572)		
	GDM (*n* = 662)	Non-GDM (*n* = 3294)	*P*	GDM (*n* = 739)	Non-GDM (*n* = 5833)	*P*
Age, years	31.73 ± 3.74	30.39 ± 3.52	< 0.001	32.24 ± 3.95	30.66 ± 3.69	< 0.001
Pre-BMI, kg/m^2^	22.63 ± 3.43	21.21 ± 2.71	< 0.001	22.45 ± 3.37	21.04 ± 2.69	< 0.001
Categorized pre-BMI			< 0.001			< 0.001
Underweight, n (%)	47 (7.10)	365 (11.80)		61 (9.06)	812 (14.53)	
Normal weight, n (%)	436 (65.86)	2513 (76.29)		434 (64.49)	4070 (72.85)	
Overweight, n (%)	128 (19.33)	348 (10.56)		127 (18.87)	557 (9.97)	
Obese, n (%)	51 (7.70)	68 (2.06)		51 (7.58)	115 (2.06)	
FH, n (%)			< 0.001			< 0.001
No	403 (60.90)	2468 (74.90)		530 (71.70)	4960 (85.00)	
Yes	259 (39.10)	826 (25.10)		209 (28.30)	873 (15.00)	
FPG, mmol/L	4.61 ± 0.45	4.42 ± 0.38	< 0.001	4.59 ± 0.52	4.38 ± 0.38	< 0.001
Parity, n (%)			< 0.001			< 0.001
Nullipara	498 (75.23)	2697 (81.88)		507 (68.61)	4337 (74.35)	
Primi or multipara	164 (24.77)	597 (18.12)		232 (31.39)	1496 (25.65)	
Educational levels, n (%)			0.314			0.074
Primary education	194 (29.31)	867 (26.32)		82 (11.10)	519 (8.89)	
Bachelor	362 (54.68)	1825 (55.40)		529 (71.58)	4173 (71.54)	
Master	99 (14.95)	557 (16.91)		111 (15.02)	1011 (17.33)	
Doctor	7 (1.06)	45 (1.37)		16 (2.30)	130 (2.23)	
Fetal sex, n (%)			0.095			0.399
Male	332 (50.15)	1535 (46.59)		345 (46.68)	2815 (48.26)	
Female	330 (49.85)	1759 (53.40)		394 (53.32)	3018 (51.74)	

Data are *n* (%), mean ± SD or median ± SD

*p* values for differences between two groups were obtained by ANOVA or χ2 test

*GDM* gestational diabetes mellitus; *pre-BMI* pre-pregnancy body mass index, *FH* family history; *FPG* fasting plasma glucose

### Logistic regression results

Table 2 shows the incidence of risk factors for GDM with OR from a multiple logistic regression model in the exploratory cohort (*n* = 3956). Increasing age, pre-pregnancy BMI, FPG and a family history of diabetes were confirmed as independent risks for GDM.

**Table 2:** GDM risk factors according to disease status and Odds Ratios (ORs) in two cohorts

Variables	Exploratory cohort (*n* = 3956)			External validation cohort (*n* = 6572)		
	Crude OR (95% CI)	Adjusted OR (95%CI)	*P*	Crude OR (95% CI)	Adjusted OR (95%CI)	*P*
Age, years	1.11 (1.08–1.14)	1.09 (1.06–1.11)	< 0.0001	1.12 (1.09–1.14)	1.01 (1.07–1.12)	< 0.0001
Pre-BMI, kg/m^2^	1.18 (1.14–1.22)	1.13 (1.09–1.17)	< 0.0001	1.17 (1.13–1.21)	1.11 (1.08–1.15)	< 0.0001
Family history	1.89 (1.57–2.26)	1.64 (1.36–1.98)	< 0.0001	2.19 (1.81–2.66)	1.93 (1.58–2.35)	< 0.0001
FPG	3.28 (2.62–4.11)	2.60 (2.07–3.27)	< 0.0001	3.47 (2.77–4.34)	2.77 (2.20–3.48)	< 0.0001
parity	1.49 (1.22–1.81)	1.14 (0.91–1.43)	0.266	1.32 (1.12–1.56)	0.89 (0.74–1.07)	0.216

Based on multiple logistic regression models adjusted mutually

*OR* odds ratio, *pre-BMI* pre-pregnancy body mass index, *FPG* fasting plasma glucose

The projected GDM risk was estimated with the equation:

$$ \mathit{\mathsf{P}}=\mathsf{1}/\left[\mathsf{1}+\mathsf{\exp}\ \left(-\mathsf{10.84}+\mathsf{0.078}\ast \mathsf{age}+\mathsf{0.119}\ast \mathsf{BMI}+\mathsf{0.893}\ast \mathsf{FPG}+\mathsf{0.491}\ast \mathsf{positive}\ \mathsf{family}\ \mathsf{history}\ \mathsf{of}\ \mathsf{diabetes}\right)\right] $$

### Development of GDM-predicting nomogram

**Fig. 1:**
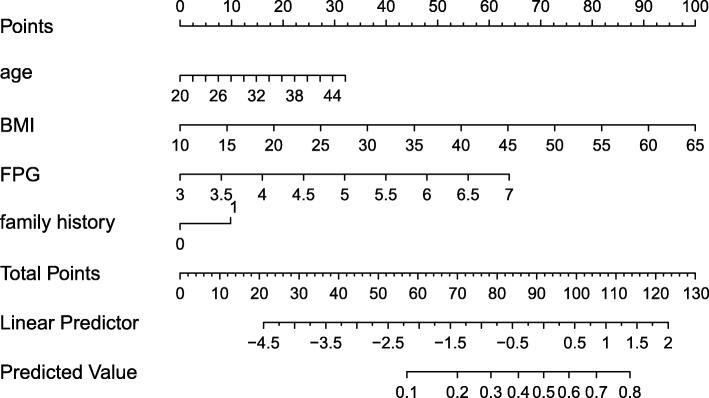
Nomogram to estimate the risk of GDM. Each predictor is assigned a score on each axis. Compute the sum of points for all predictors and denote this value as the total points. The corresponding “risk of GDM” of “total point” was converted to a predicted probability of GDM

A nomogram incorporating age, pre-pregnancy BMI, FPG in the first trimester and a family history of diabetes was developed and presented as shown in Fig. 1.

### Discrimination results

We depicted ROC curves with 3956 subjects from the exploratory cohort. The AUC for the prediction nomogram was 0.69 [95% CI:0.67–0.72, *p* < 0.0001], while the corrected c-index was 0.69 in the internal bootstrap validation. Applying the exploratory set estimates to the validation set yielded a similar AUC of 0.70 [95% CI: 0.68–0.72, *p* < 0.0001].

### Calibration results

**Fig. 2:**
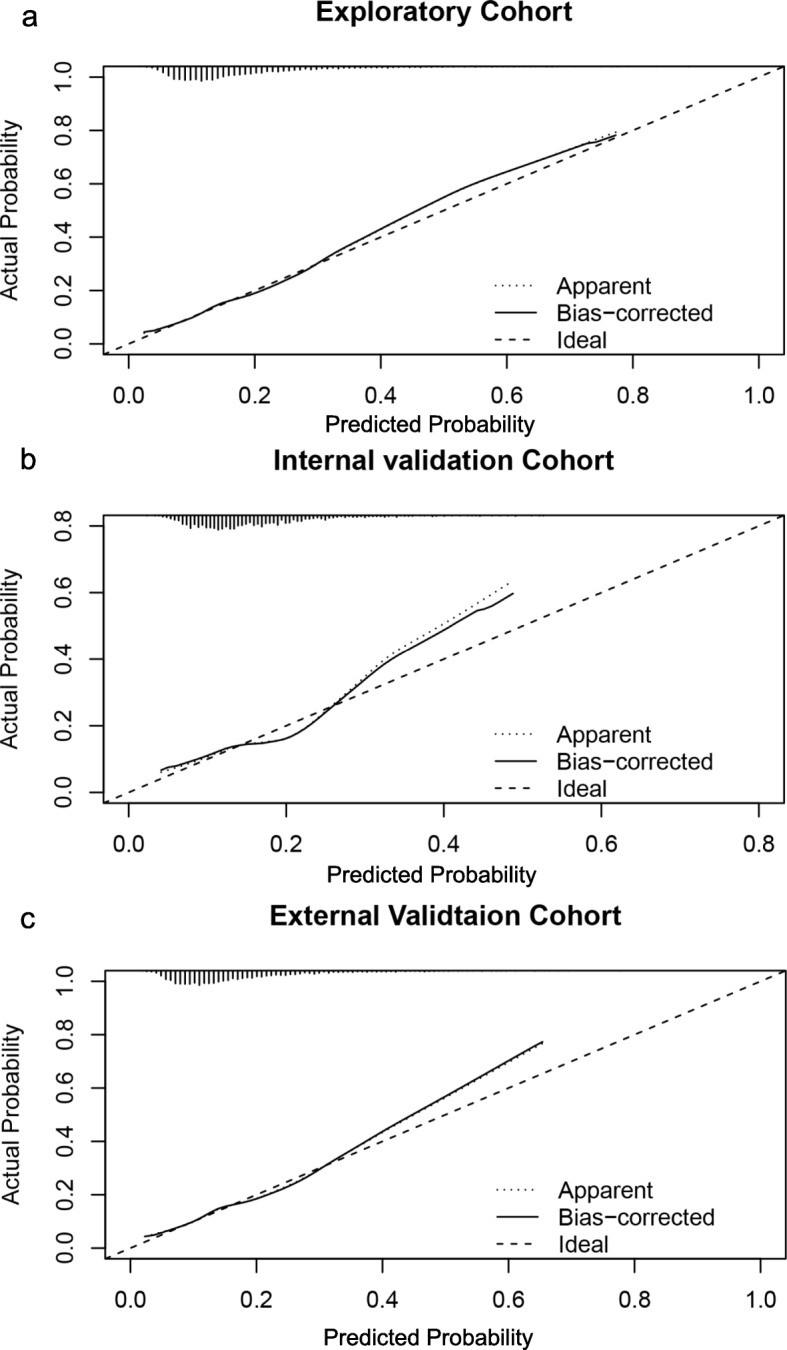
Calibration results. Nomogram-predicted probability of GDM is plotted on the x-axis; actual probability of GDM is plotted on the y-axis. The ideal calibration line means an intercept of 0 and a slope of 1 for the calibration plot. Exploratory cohort (**a**); Internal validation cohort (**b**); External validation cohort (**c**)

The probability of GDM occurrence in the exploratory and validation cohorts was accurately predicted form the calibration curve (Fig. 2a-c).

### Clinical use

**Fig. 3:**
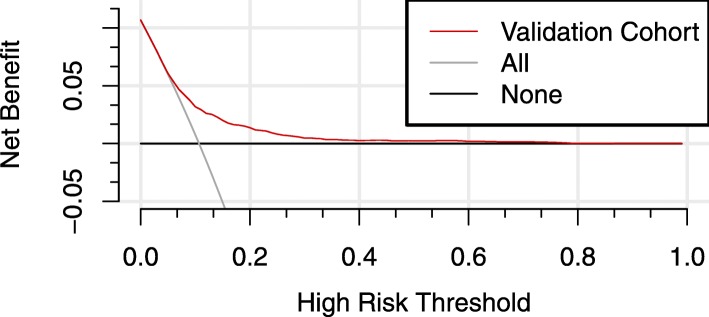
Decision curve analysis for gestational diabetes mellitus. Solid black line = net benefit when no one is at risk for gestational diabetes mellitus (GDM); grey line = net benefit when all are at risk for GDM. The y-axis measures the net benefit. The red line represents the nomogram. The decision curve showed that if the threshold probability is between 0.05–0.78, using the nomogram in the current study to predict GDM adds more benefit than the intervention-all-patients scheme or the intervention-none scheme

For predicted probability thresholds between 0.05 and 0.78, our model showed a positive net benefit, without increasing the number of false positives (Fig. 3).

### Screening ability

**Fig. 4:**
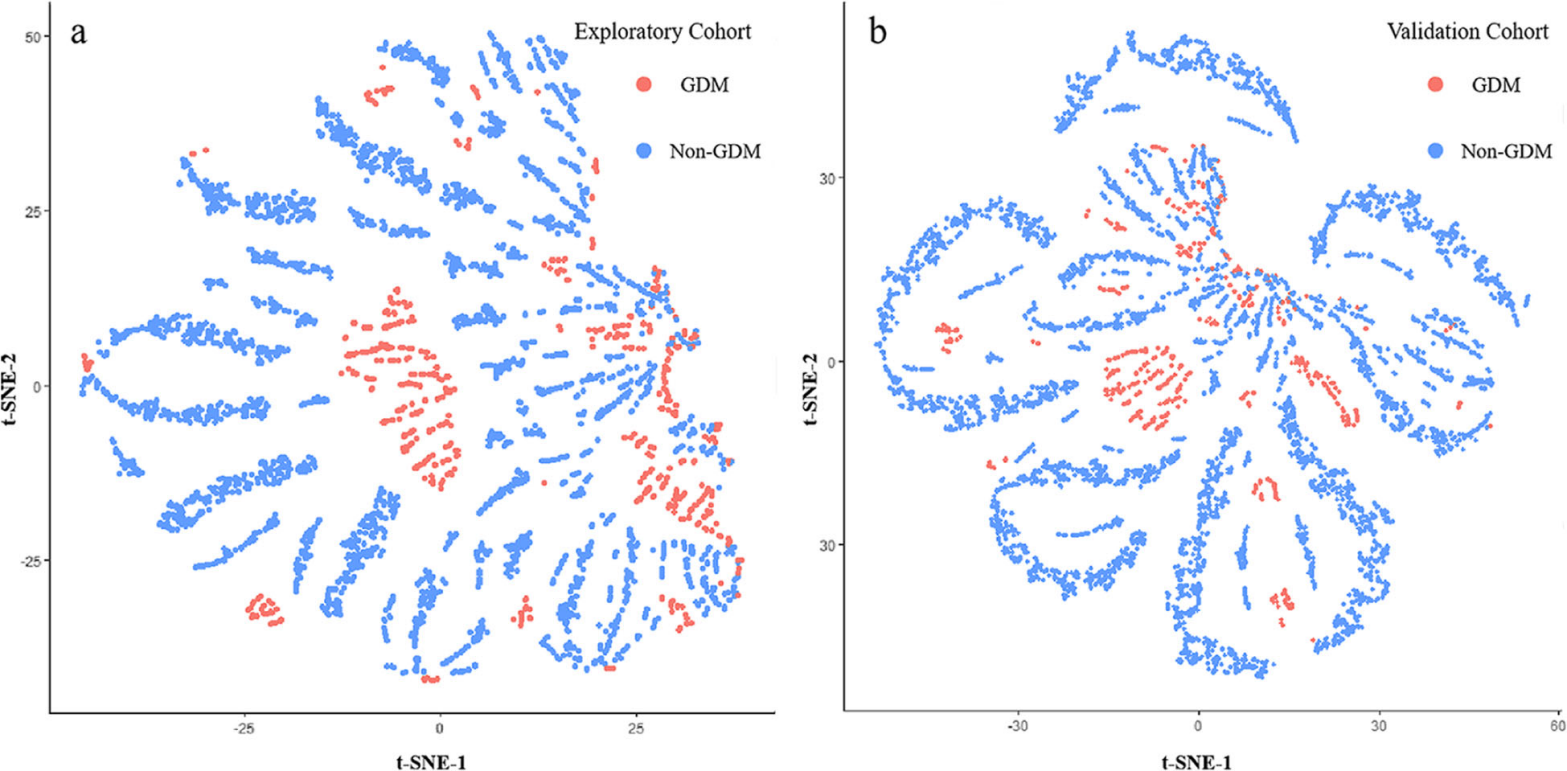
Screening ability of this model. Orange color represented for GDM women, blue point represented for non-GDM women. t-SNE result showed the majority of non-GDM women were separated from GDM women by an obvious boundary in the retrospective cohort (**a**). t-SNE showed the similar result in the prospective cohort (**b**). GDM: gestational diabetes mellitus

To assess the suitability of linking the structure revealed by t-SNE to clinical outcome, and thereby discriminating subpopulations, we converted the t-SNE space to a*b color space. In the resulting t-SNE image obtained by density-based analysis, each pixel was colored according to its property (Orange for GDM and blue for non-GDM). In both cohorts (Fig. 4a-b), the distinct regions of GDM and non-GDM outlined by t-SNE were separated, hence generating transitional boundaries that could be highlighted by two different colors. This demonstrated the ability to distinguish GDM from non-GDM women.

## Discussion

In this study, we developed and validated a diagnostic nomogram for the prediction of GDM. The nomogram incorporated four risk factors including age, pre-pregnancy BMI, FPG in the first trimester and history of diabetes in first-degree relatives. Earlier in pregnancy, the nomogram successfully stratified the women according to their risk of developing GDM. Its prediction was supported by the AUC (0.69 and 0.70 for the exploratory and validation cohorts, respectively) and the calibration curve. Incorporation of the easily accessible risk factors into our nomogram enhanced the process individualized prediction of GDM.

Heterogeneity of physiological processes underlying hyperglycemia has been reported among women with GDM [19]. Therefore, an integrated approach that combines multiple risk indicators, could accurately predict the women who should be screened further for GDM. FPG widely used as a screening test for GDM through detection of preexisting diabetes (FPG ≥ 7 mmol/L) in the first trimester. High pre-pregnancy BMI or normal upper range of FPG was associated with insulin sensitivity defect, condition which could be involved in the onset of GDM [5, 19]. In China, it is traditional for women to increase their calorie consumption and sedentary habit from the onset of pregnancy though this is clearly responsible for increasing diabetogenic burden in high risk women [20]. It should be emphasized that our study was conducted at a time when early identification and targeted interventions are available for those women .

Various first-trimester prediction models for GDM have been proposed [21–23]. However, these models are not commonly used in routine clinical care. First, this could be partly attributed to poor external validation of these models since clinical use was not considered during their development. Secondly, indicators, such as high molecular weight adiponectin, omentin-1 and interleukin-6, yield a high AUC, but also increase the psychological and economic burden [24]. We generated a decision curve analysis to quantify the clinical usefulness, and a key finding was that a single probability threshold could be used both to categorize patients as positive or negative, and to weight false positive and false negative classifications [25]. In our model, the use of a 0.05–0.78 threshold to identify individuals with high risk of GDM always had a positive net benefit.

HbA1c showed a high sensitivity but insufficient power to diagnose GDM [26]. Sumaiya Adam et al. [22] reported that adding a HbA1c did not significantly improve the predictive value of prediction model for GDM. In addition, HbA1c levels vary with gestational hemodilution, hemoglobin disease, and/or anemia. Therefore, we did not consider it as risk factor, in our model.

To assess its predictive performance, we validated our model both internally and externally. Its main advantage is the ability to integrate information that is routinely obtained at the first antenatal visit into a graphical nomogram. Heterogeneity was observed between the demographic characteristics of the train set and external validation set. Therefore, heterogeneity could be used to reflect and evaluate the applicability of the model in external population, and improve the overall application value of the model. Inclusion of both first and second degree relatives with diabetes in the retrospective cohort could have over-estimated the contribution of family history with some measurement bias. Hence, in the prospective cohort we only considered the first degree relatives with diabetes.

This study had the following limitations. First, our study was based on the hospital cohort, and the homogeneity of the cohort increased the internal validity while wakening the confidence of representation of the Chinese population. A prospective, multicenter, trial could therefore be required to test our prediction model. Second, we did not incorporate excessive gestational weight gain though it was a key factor in GDM development. Third, the sample size of the retrospective cohort was smaller than the prospective cohort, due to some women returning to their home towns to deliver, while others refused to participate in the study in 2015. However, the final sample size (*n* = 3956) was sufficient for establishing the prediction model, indicating that these attritional features did not result in a type I error. Finally, the AUC value in our model did not show high prediction accuracy. However, since AUC metric solely focuses on the predictive accuracy. It cannot be used to identify a preferable model. A model with a greater specificity but slightly lower sensitivity would have a higher AUC, but would be a poorer choice for clinical use. We therefore further used Decision-analytic methods, which incorporated our results and theory to determine the worthiness of a model or alternatives.

## Conclusions

In conclusion, we have developed a simple nomogram for pregnant Chinese women that can be used to estimate the probability of developing GDM at the first antenatal visit. This prediction model could identify women at risk for GDM early in pregnancy, allowing for timely intervention to improve maternal outcome. However, further studies should be carried out in other cities to improve the prediction accuracy of GDM risk in the Chinese, or even Asian populations.

## Data Availability

The datasets used and/or analysed during the current study are available from the corresponding author on reasonable request.

## Abbreviations

ADA: American Diabetes Association
AUC: Area under the curve
BMI: Body mass index
CI: Confidence interval
FPG: Fasting plasma glucose
GDM: Gestational of diabetes
HbA1c: Glycated hemoglobin
IPMCH: International Peace Maternity and Child Care Health Hospital
OGTT: Oral glucose tolerance test
OR: Odds ratio
ROC: Receiver operating characteristic
t-SNE: t-distributed stochastic neighbor embedding
